# Independent regulation of strigolactones and blumenols during arbuscular mycorrhizal symbiosis in rice

**DOI:** 10.1111/tpj.16848

**Published:** 2024-05-31

**Authors:** Emily K. Servanté, Rayko Halitschke, Catarina Rocha, Ian T. Baldwin, Uta Paszkowski

**Affiliations:** ^1^ Crop Science Centre, Department of Plant Sciences University of Cambridge Cambridge UK; ^2^ Max Planck Institute for Chemical Ecology (MPI CE) Jena Germany

**Keywords:** arbuscular mycorrhizal symbiosis, rice, apocarotenoids, blumenol‐C‐glucosides, strigolactones

## Abstract

The apocarotenoid strigolactones (SLs) facilitate pre‐symbiotic communication between arbuscular mycorrhizal (AM) fungi and plants. Related blumenol‐C‐glucosides (blumenols), have also been associated with symbiosis, but the cues that are involved in the regulation of blumenol accumulation during AM symbiosis remain unclear. In rice, our analyses demonstrated a strict correlation between foliar blumenol abundance and intraradical fungal colonisation. More specifically, rice mutants affected at distinct stages of the interaction revealed that fungal cortex invasion was required for foliar blumenol accumulation. Plant phosphate status and D14L hormone signalling had no effect, contrasting their known role in induction of SLs. This a proportion of the SL biosynthetic enzymes, D27 and D17, are equally required for blumenol production. These results importantly clarify that, while there is a partially shared biosynthetic pathway between SL and blumenols, the dedicated induction of the related apocarotenoids occurs in response to cues acting at distinct stages during the root colonisation process. However, we reveal that neither SLs nor blumenols are essential for fungal invasion of rice roots.

## Significance Statement

The apocarotenoids strigolactone and blumenol are both associated with arbuscular mycorrhizal symbiosis. Servanté et al. report divergent regulation of each compound despite the partially shared biosynthetic pathway, thereby highlighting the involvement of distinct tissues and signalling cues at different stages of the interaction.

## INTRODUCTION

Arbuscular mycorrhizal fungi (AMF) of the phylum Glomeromycotina form intimate associations with >80% land plants today and significantly facilitate the plant's uptake of minerals and water from the soil. Following reciprocal recognition in the rhizosphere, fungal hyphae penetrate host roots and form intracellular arbuscules in inner cortical cells. At these highly branched structures, AMF provide predominantly inorganic phosphate (P_i_) to the plant, in return for organic carbon (Smith & Read, 2008).

Over the past two decades, genetic studies have identified a suite of plant genes required for functional establishment of the symbiosis. The common symbiosis signalling pathway (CSSP) is equally required for legume interaction with AMF and nitrogen‐fixing rhizobia and is equivalently necessary for enabling AM fungal root colonisation in non‐legumes. Mutants of CSSP genes consistently show abnormal fungal penetration structures and often limited colonisation of the outer root cell layers (Gutjahr et al., 2008). In an additional signalling pathway, activation of the DWARF14‐LIKE (D14L) receptor leads to the suppression of the negative regulator SMAX1, which in turn de‐represses genetic programmes underpinning AM symbiosis development in rice, including CSSP components (Choi et al., 2018; Gutjahr et al., 2015).

Early rhizosphere signalling commences with plant strigolactones (SLs), apocarotenoid phytohormones, which show pronounced production and secretion in roots of P_i_ starved plants and act as signalling molecules to activate AMF for symbiosis, reflected by enhancing spore germination, hyphal branching, mitosis, respiration and growth (Akiyama et al., 2005; Besserer et al., 2006, 2008; Yoneyama, Xie, et al., 2007; Yoneyama, Yoneyama, et al., 2007). Mutants of SL biosynthesis genes in rice have reduced fungal root colonisation but support the formation of morphologically wild‐type fungal structures (Gutjahr et al., 2012; Kobae et al., 2018). Interestingly, the D14L signalling pathway regulates both SL biosynthesis and AM symbiosis and thereby integrates attraction of the fungus with conditioning plants for AM symbiosis (Choi et al., 2020; Hull et al., 2021).

Blumenols (C_13_ cyclohexenone derivatives) and yellow‐pigmented mycorradicin (C_14_ polyene derivatives) are also apocarotenoids that are long known to accumulate in roots colonised by AMF (Klingner et al., 1995; Maier et al., 1995). Foliar accumulation of distinct blumenol‐C‐glucosides was found to positively correlate with AMF root colonisation and is thereby suitable as foliar markers for root colonisation in the assessed diverse plant species (Wang et al., 2018). Despite this, little is known of the role blumenol plays during symbiosis.

The biosynthesis of apocarotenoids involves carotenoid cleavage, which can be achieved either non‐enzymatically by reactive oxygen species or through catalysis by carotenoid cleavage dioxygenase enzymes (CCDs) (Fiorilli et al., 2019). SL biosynthesis has been well defined in rice, and genetic studies have identified multiple enzymatic reactions. First, reversible isomerisation of β‐carotene to 9‐cis‐β‐carotene (9‐cis‐C40) occurs via iron‐binding β‐carotene isomerase, DWARF 27 (D27) (Abuauf et al., 2018; Alder et al., 2012). This product is cleaved to 9‐cis‐β‐apo‐10′‐carotenal (9‐cis‐C27) by the stereospecific CCD7/D17 (Alder et al., 2012; Bruno et al., 2014). This is further cleaved by CCD8/D10 to form carlactone (CL), the biosynthetic precursor for SLs (Alder et al., 2012; Bruno et al., 2017). Although information on blumenols is more limited, studies using *ccd7* mutants in tomato and peas elucidated involvement of CCD7 in the first cleavage step in blumenol biosynthesis (López‐Ráez et al., 2015; Vogel et al., 2010; Walter et al., 2010). Current models assume CCD7 catalyses cleavage of the C40 isomer to a C13 cyclohexenone and a C27 apocarotenoid. The C27 intermediate is subsequently cleaved by CCD1 to yield a second C13 cyclohexenone (Floss et al., 2008; Vogel et al., 2010; Walter et al., 2010). AMF‐specific blumenol‐C‐glucosides are then likely formed from C13 cyclohexanones by the action of yet unknown cytochrome P450s and glycosyltransferases (You et al., 2023). Notably, an additional SL biosynthesis enzyme, D27, has been suggested to facilitate C40 isomerisation required for substrate specificity of CCD7 (Bruno et al., 2014), but no direct evidence for a role in blumenol biosynthesis has yet been reported (Fiorilli et al., 2019; Wang et al., 2018).

Here, we establish leaf blumenol as a reliable marker for fungal root colonisation in rice and use available rice signalling mutants to pinpoint the stage of AM symbiosis development at which blumenol accumulation occurs. We further elucidate independence of blumenol accumulation from rhizospheric signalling processes that regulate SLs, such as plant‐phosphate status and D14L/SMAX1 signalling, and provide the first direct evidence for accumulation being strictly associated with cortical invasion and arbuscule formation. Despite SLs and blumenol occurrence at distinct stages of symbiosis, we provide direct evidence for a partially shared biosynthetic pathway. We interrogate AM phenotypes in mutants with abolished SLs and blumenol accumulation (*d27* and *d17*) and abolished SL accumulation only (*d10*) and clarify that neither blumenols nor SLs are essential for development of AM fungal root invasion.

## RESULTS

### Identification of AM symbiosis‐indicative blumenol‐C‐glucosides in rice

**Figure 1:**
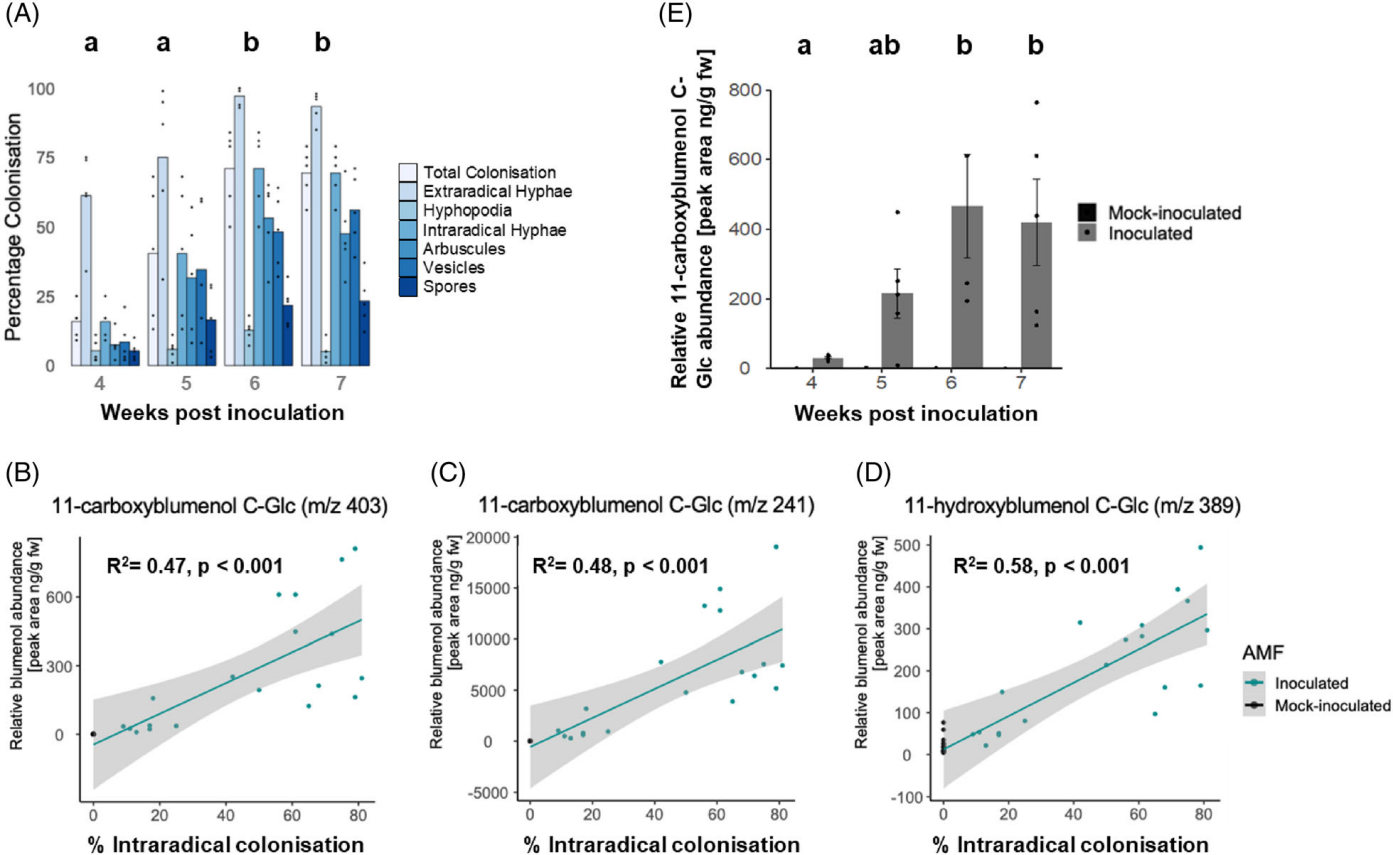
Accumulation of blumenol C‐glucosides during progressive arbuscular mycorrhizal (AM) symbiosis development in rice. (A) Bars represent average percentage colonisation by AM fungal structures at 4, 5, 6 and 7 weeks post‐inoculation (wpi) with 300 *Rhizophagus irregularis* spores per plant. *P* < 0.05 for total intraradical colonisation, Kruskal–Wallis followed by *post‐hoc* testing. (B–D) Linear regression between percentage intraradical colonisation [total colonisation in (A)] and foliar abundance of blumenol‐C‐glucoside derivatives; (B) 11‐carboxyblumenol C‐Glc [*m*/*z* 403, molecular ion [M + H]+ (B); *m*/*z* 241, aglycon precursor ion [M + H‐Glc]+ (C)] and (D) 11‐hydroxyblumenol C‐Glc (*m*/*z* 389, molecular ion [M + H]+). *R* ^2^ and *P*‐value of regression are noted, and grey area represents 95% confidence intervals. (E) Relative abundance of arbuscular mycorrhizal fungi (AMF)‐specific derivative 11‐carboxyblumenol C‐Glc in shoot material from the same samples as (A). Bars represent average ± SE. *P* < 0.05, AMF‐inoculated plants, Kruskal–Wallis test followed by *post‐hoc* testing. Same letters indicate no significant difference, different letter indicate significant difference.

Blumenol‐C‐glucosides have been reported as strict foliar markers of root colonisation in different plant species, but were not described for rice (Wang et al., 2018). To determine the correlation of root colonisation with accumulation of specific foliar blumenol derivatives in rice, we conducted a time‐course experiment in *Oryza sativa* cv. Nipponbare inoculated with *Rhizophagus irregularis* and assessed both the extent of fungal colonisation of the root and the abundance of blumenol C‐glucosides in the leaf. We observed an increase in the quantity of all extra‐ and intraradical fungal structures from 4 to 6 weeks post‐inoculation (wpi), which then reached a plateau and stayed largely unchanged at 7 wpi (Figure 1A). Examining foliar blumenols, we found that 11‐carboxy‐ and 11‐hydroxyblumenol  C‐glucoside abundance correlated with the percentage of total root colonisation in rice (Figure 1B–D). While we observed some accumulation of 11‐hydroxyblumenol C‐glucoside in mock‐inoculated plants (Figure 1D), 11‐carboxyblumenol C‐glucoside accumulated strictly in AMF‐inoculated plants (Figure 1B,C). We therefore conclude that 11‐carboxyblumenol C‐glucoside (from now on blumenol) is a suitable foliar marker for rice root colonisation of *O. sativa* cv. Nipponbare (Figure 1E).

### Blumenol‐C‐glucoside accumulation in leaves strictly associates with AM fungal cortex colonisation in rice

**Figure 2:**
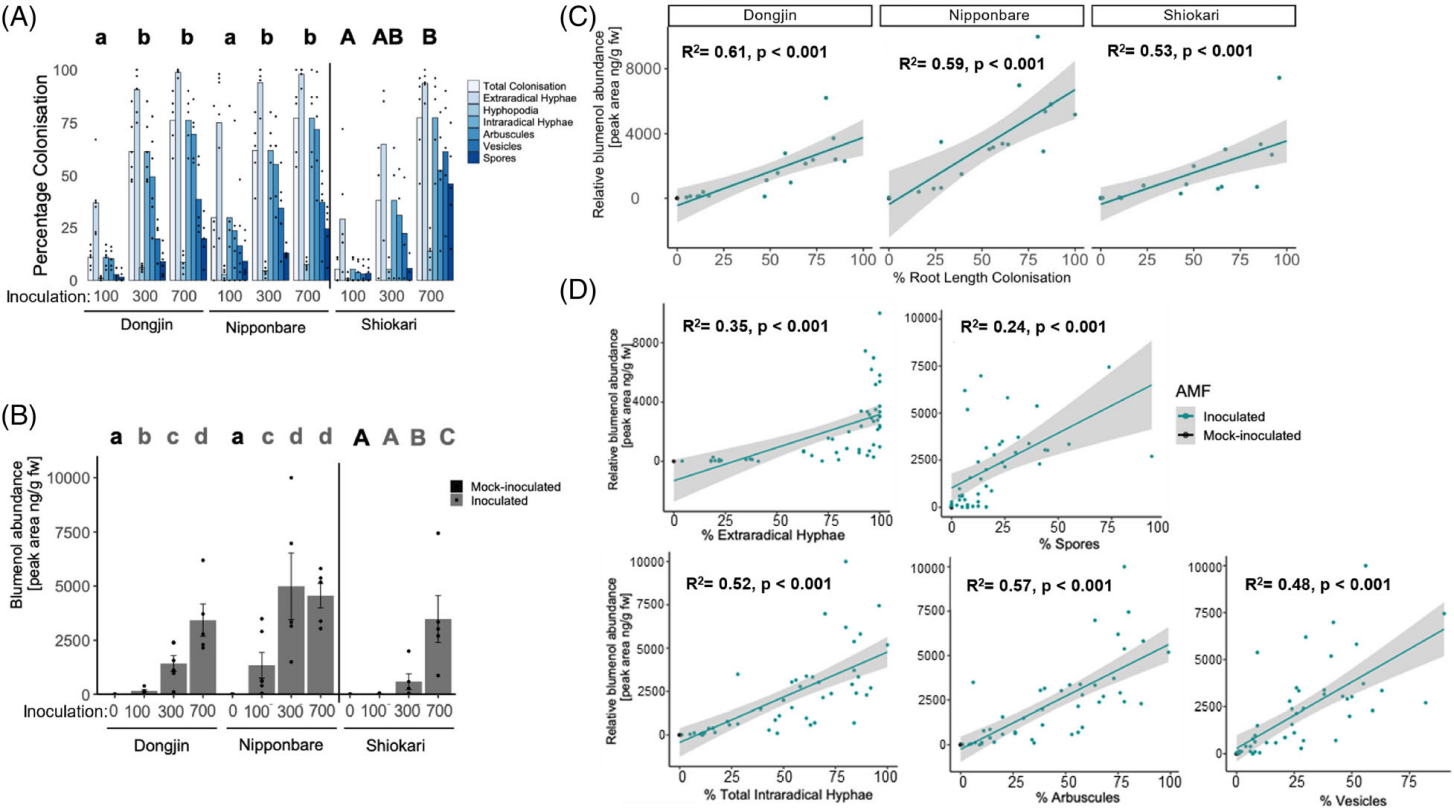
Dynamics of blumenol accumulation during arbuscular mycorrhizal (AM) symbiosis in rice. (A–D) *Oryza sativa* subsp. *japonica* cultivars inoculated with 100, 300 or 700 *Rhizophagus irregularis* spores per plant. *Nipponbare* and *Dongjin* were analysed at 6 weeks post‐inoculation (wpi) and *Shiokari* at 9 wpi. *n* = 4–6. (A) Bars represent average percentage colonisation by AMF life‐cycle structures indicated. *P* < 0.05 for total intraradical colonisation. (B) Relative blumenol accumulations in shoot material from the same samples. Bars represent average ± SE. *P* < 0.05, mock‐ (black letter) and AMF‐inoculated (grey letter). (A, B) Kruskal–Wallis test followed by *post‐hoc* testing. (C, D) Linear regression between foliar blumenol abundance and percentage (C) intraradical colonisation per cultivar or (D) AMF structures indicated. *R* ^2^ and *P*‐value of regression are noted, and grey area represents 95% confidence intervals.

To establish the robustness of blumenol in the leaf as an indicator for the extent of fungal root colonisation, we used three model *O. sativa* subsp. *japonica* cultivars, Nipponbare, Dongjin and Shiokari, and conducted an *R. irregularis* inoculation gradient experiment. As expected, we found that increased inoculum strength led to enhanced root colonisation (Figure 2A), and similarly to foliar accumulation of blumenol (blumenol, Figure 2A–D). We thereby confirmed suitability of blumenol as a robust leaf marker for AM symbiosis establishment in the root across diverse rice cultivars.

Interestingly, the correlation between root colonisation and leaf blumenol accumulation was most significant for intraradical colonisation structures, which collectively include hyphae, arbuscules and vesicles compared to extraradical structures (Figure 2D). To better understand at which stage of the interaction an accumulation of blumenol occurred, we used rice signalling mutants, perturbed at different stages of the interaction, namely *CHITIN ELICITOR RECEPTOR KINASE 1* (*CERK1*), *POLLUX*, *CALCIUM/CALMODULIN‐DEPENDENT KINASE* (*CCAMK*) and *STUNTED ARBUSCULES 1* (*STR1*). *CERK1* encodes a bi‐functional LysM receptor‐like kinase, and root colonisation is reduced but morphologically normal in rice *cerk1* mutants (Miyata et al., 2014). Following fungal perception, the nuclear ion‐channel POLLUX and calcium/calmodulin‐dependent kinase CCAMK are required for generation and deciphering of symbiotic calcium signatures (Choi et al., 2018), enabling cortex invasion and arbuscule formation, which is lost in the corresponding rice knock‐out (KO) mutants (Gutjahr et al., 2008). STR1 is a half‐size ABC transporter involved with lipid export to AMF and *str1* mutants form stunted arbuscules (Gutjahr et al., 2012; Zhang et al., 2010).

**Figure 3:**
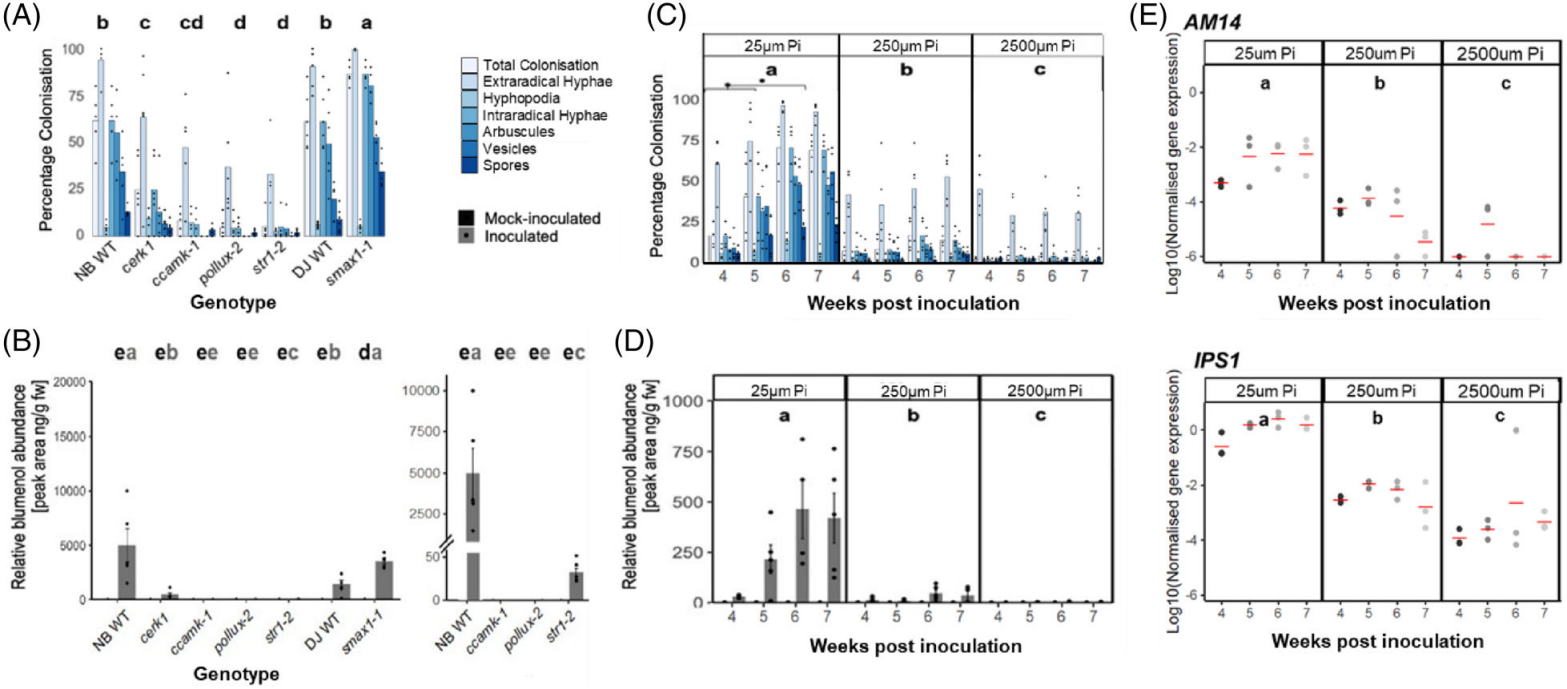
Regulation of blumenol accumulation during arbuscular mycorrhizal (AM) symbiosis in rice. (A, B) AM symbiosis mutants and corresponding *Nipponbare* (NB WT) and *Dongjin* (DJ WT) wild‐type cultivars at 6 weeks post‐inoculation (wpi) with 300 *Rhizophagus irregularis* spores/plant. *n* = 4–6. (A) Bars represent average percentage colonisation by arbuscular mycorrhizal fungi (AMF) life‐cycle structures indicated. *P* < 0.05 for total intraradical colonisation. (B) Relative blumenol accumulations in shoot material from the same samples (left), with zoomed, broken axis for visualisation of lower abundance (right). Bars represent average ± SE. *P* < 0.05, mock‐ (black letter) and AMF‐inoculated (grey letter). (C–E) Time‐resolved phosphate (P_i_) gradient experiment at 4–7 wpi with 300 *R. irregularis* spores/plant, *n* = 4–6. (C) Bars represent average percentage colonisation by AMF life‐cycle structures indicated. *P* < 0.05 for total intraradical colonisation in P_i_ treatment. (D) Relative blumenol accumulations in shoot material from the same samples. Bars represent average ± SE. *P* < 0.05, and letters represent statistical differences between P_i_ treatment and asterisks differences between wpi. (E) Transcript accumulation of late AM marker *AM14* and phosphate starvation marker *IPS1*, representing gene expression GEOmean normalised to *OsCYCLOPHILIN*. Data points of three biological replicates, each with three technical replicates. Red bar indicates mean, and colour indicates wpi. *P* < 0.05 for transcript accumulation per P_i_ treatment. (A–E) Kruskal–Wallis followed by *post‐hoc* testing.

*R. irregularis*‐inoculated *cerk1* had reduced intraradical colonisation (Figure 3A), which was mirrored by reduced blumenol accumulation (Figure 3B). This suggests that *CERK1* is not required for blumenol accumulation. In inoculated *pollux* and *ccamk*, intraradical colonisation was severely reduced with no arbuscule formation (Figure 3A). Foliar blumenol accumulation was undetectable (Figure 3B), corroborating previous reports of abolished blumenol accumulation in *Nicotiana attenuata CCamK* knock‐down mutants (Wang et al., 2018). Root colonisation in *R. irregularis*‐inoculated *str1* was reduced to similar severity as observed in *pollux* and *ccamk* but stunted arbuscules occurred at low frequency (Figure 3A). Foliar blumenol abundance was also strongly reduced (Figure 3B, left), but still detectable (Figure 3B, right), maintaining correlation with arbuscule formation, despite their shrivelled morphology. The maintenance of blumenol accumulation in *str1* but not *pollux* and *ccamk*, despite similarly reduced overall levels of colonisation, provides strong support that colonisation of the root surface and outer root cell layers is insufficient to stimulate blumenol production. We therefore conclude that blumenol accumulation is strictly dependent on cortex colonisation and possibly intracellular arbuscule formation.

### Accumulation of AM symbiosis‐indicative blumenol is independent of early rhizospheric signals and phosphate status

The α/β hydrolase DWARF14‐LIKE (D14L) has an essential function in enabling AM symbiosis in rice, acting as an ‘on–off’ switch where D14L forms a complex with E3 ubiquitin ligase DWARF3 (D3) to facilitate removal of the negative regulator SUPPRESSOR OF MAX2 1 (SMAX1) to enable root colonisation. Genetic programmes for both SL biosynthesis and AM symbiosis are induced when functional SMAX1 is absent, facilitating pre‐symbiotic communication (Choi et al., 2020). Conceivably, removal of SMAX1 could not only lead to the increased biosynthesis of SL but also of blumenol. We were however unable to consistently detect blumenol in non‐inoculated *smax1* plants (Figure 3B). Thus, while the D14L/SMAX1 pathway is sufficient for induction of SLs (Choi et al., 2020), it is not sufficient for the production of blumenol. *R. irregularis*‐inoculated *smax1* showed enhanced AM fungal root colonisation and correlating increased shoot blumenol quantities compared to wild‐type (Figure 3A,B), confirming the tight requirement of root colonisation for leaf blumenol accumulation.

Arbuscular mycorrhizal symbiosis and SL biosynthesis are additionally regulated by the P_i_ status of the plant, (reviewed in Paries & Gutjahr, 2023). To verify if blumenol accumulation can be stimulated by the host P_i_ status, we conducted a time‐resolved P_i_‐dose experiment and confirmed reduced root colonisations with increasing P_i_ fertilisation regime (Figure 3C, E top). Foliar blumenol was undetectable in mock‐inoculated plants at any phosphate concentration but mirrored the extent of AM symbiosis development in *R. irregularis*‐inoculated plants (Figure 3D). Plant P_i_ starvation at 25 μm P_i_ was confirmed by transcript accumulation of a marker of phosphate starvation, *INDUCED BY PHOSPHATE STARVATION 1* (*IPS1*) (Hou et al., 2005), which was increased in plants grown in 25 μm compared to 250 and 2500 μm P_i_ respectively (Figure 3E bottom). Thus, foliar blumenol accumulation was tightly associated with root colonisation, independent of the plant P_i_ nutrient status. Together, we report that blumenol accumulation is independent of regulation by SMAX1‐ or nutrient signalling mechanisms that facilitate SL production during early rhizosphere communication.

### Both D27 and D17/CCD7 are required for blumenol biosynthesis in rice

**Figure 4:**
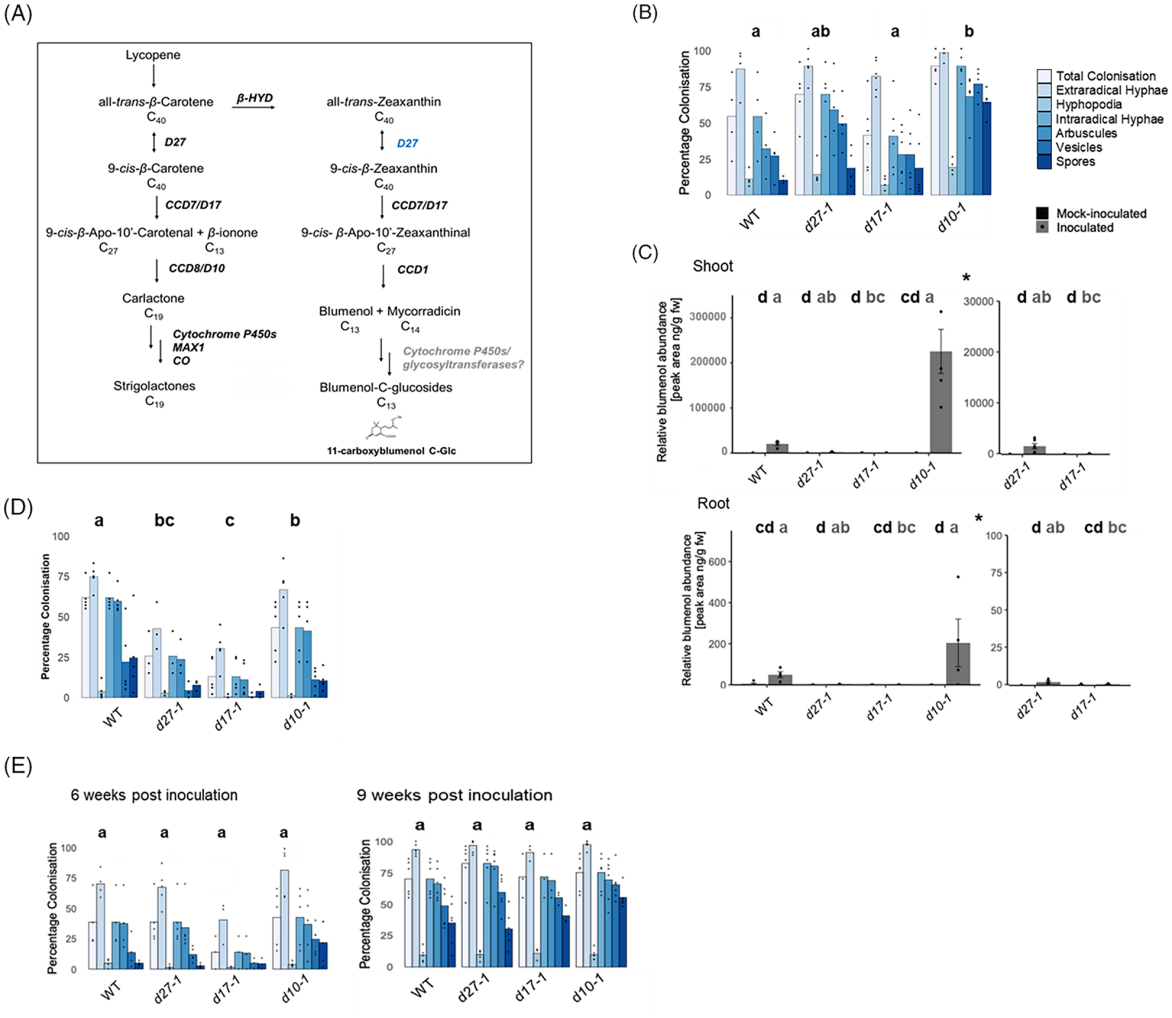
Characterisation of the role of strigolactone (SL) and blumenol biosynthesis genes in rice AM symbiosis. (A) Current model of blumenol and SL biosynthesis in rice, modified from Wang et al. (2018) and Fiorilli et al. (2019). SLs and blumenol apocarotenoids are produced by cleavage dioxygenase enzymes (CCDs) and modifying enzymes, depicted in black boldface if identity previously confirmed, grey boldface if not and blue for new evidence presented in this study. β‐HYD, β‐hydroxylase; CYP97A, heme‐containing cytochrome P450 carotene β‐ring hydroxylase; D27, DWARF27; CCD7/D17, DWARF17; CCD8/D10, DWARF10; and MAX1, MORE AXILLARY GROWTH1. (B–E) SL biosynthesis mutants (cv. *Shiokari*), inoculated with *Rhizophagus irregularis*, *n* = 4–6 (B) Bars represent average percentage colonisation by AMF life‐cycle structures indicated. 500 spores/plant at 6 weeks post‐inoculation (wpi). *P* < 0.05 for total intraradical colonisation. (C) Relative blumenol accumulations in shoot and root material from the same samples as (B). Bars represent average ± SE. *P* < 0.05, mock‐ (black letter) and AMF‐inoculated (grey letter). Asterisk represents zoomed axis for visualisation of lower abundances. (D) Time course experiment, 500 *R. irregularis* spores/plant analysed at 6 and 9 wpi, *n* = 3–6. Bars represent average percentage colonisation by AMF life‐cycle structures indicated. *P* < 0.05 for total intraradical colonisation. (E) SL biosynthesis mutants (cv. *Shiokari*) inoculated with 300 *R. irregularis* spores/plant at 6 weeks post‐inoculation (wpi), *n* = 4–6. (B–E) Kruskal–Wallis followed by *post‐hoc* testing.

Apocarotenoids such as SLs and blumenols are produced in roots through the cleavage of carotenoids by CCDs. A dual role of the enzyme CCD7/D17 in SL and blumenol biosynthesis has been reported in tomato (Vogel et al., 2010). It has additionally been suggested that D27, an isomerase involved in SL biosynthesis, is required (Figure 4A), although no metabolic evidence has been described from any plant species (Fiorilli et al., 2019). We investigated the requirement of SL biosynthesis genes for blumenol production in inoculated rice mutants of *D27*, *CCD7/D17* and *CCD8/D10* (Lin et al., 2009; Umehara et al., 2008). Unexpectedly, root colonisation of mutant roots was at wild‐type level or even higher at 6 wpi (Figure 4B), documenting that none of the three genes are required for symbiosis establishment and contradicting earlier reports (including our own) for *d10* and *d17* (Gutjahr et al., 2012; Kobae et al., 2018). Foliar blumenol accumulation correlated with increased root colonisation in wild type and *d10* (Figure 4C), but was absent in *d17* and *d27* (Figure 4C). Similar to shoots, blumenol accumulation was also severely reduced in roots of *d27* and *d17* plants (Figure 4C), demonstrating that the absence of blumenol in the shoot could not be explained by the impairment of systemic transport. We therefore report that, while D10 is required for SL biosynthesis only, D27 and D17 have dual roles in SL and blumenol biosynthesis (Figure 4A).

### SLs and blumenols are not critical for maintenance of root colonisation in rice

The high colonisation levels in *d17* and *d10* are perplexing and unlike previous reports of reduced colonisation in the same SL‐deficient rice mutant alleles (Gutjahr et al., 2012; Kobae et al., 2018), suggesting that the root colonisation phenotype may be more plastic. Since SLs provide positional information to the fungus, we reasoned that in their absence hyphal root encounter is random and hence more likely at either higher inoculum strength or prolonged co‐cultivation. We challenged both hypotheses and first conducted an experiment with lower inoculum strength of 300 as opposed to 500 spores in the *d27*, *d17* and *d10* mutants, harvested at the same time post‐inoculation. AM colonisation was reduced in all mutants and most severely in *d17* compared to *d27* and *d10* (Figure 4E). This observation lends support for a lower rate of hyphal root encounter in the SL‐deficient mutants explaining the reduced colonisation compared to when higher inoculum strength was used (Figure 4B). To resolve when colonisation levels would plateau in each genotype, we inspected colonisation at 6 wpi and following prolonged co‐cultivation at 9 wpi. While the *d27* and *d10* mutants at 6 wpi already had wild‐type colonisation levels, *d17* showed a tendency for lower colonisation (Figure 4D). At 9 wpi, all genotypes showed equivalent wild‐type colonisation (Figure 4D). This observation is corroborated by previous reports that, once established, colonisation development is not supressed in SL‐deficient mutants (Kobae et al., 2018). We therefore reconcile variability of root colonisation phenotypes across experiments with the fact that all mutants can eventually reach wild‐type levels of colonisation, and reduced colonisation is only observed when experiments are sampled before the ‘catch up’ at plateau occurs. Importantly, we consistently found that *d27* and *d17* mutants could sustain wild‐type levels of colonisation, arguing against a critical requirement for either SLs or blumenols in maintenance of the symbiosis.

## DISCUSSION

Previous studies have reported foliar accumulation of AM symbiosis‐associated hydroxy‐ and carboxy‐blumenol C‐glucosides in different plant species (Wang et al., 2018). We newly report that in rice, blumenol strictly accumulated during AM symbiosis. The correlation between root colonisation and foliar blumenol accumulation was similar to that described in *N. attenuata* and advocates for similar suitability as a high‐throughput foliar marker of root colonisation (Mindt et al., 2019; Wang et al., 2018).

We used available rice mutants to genetically dissect the stages where AM symbiosis development induces blumenol and reveal a requirement for cortex colonisation. This finding validates previous reports of strong correlation of blumenol accumulation with intraradical colonisation structures such as arbuscules and vesicles (Fester et al., 2002; Wang et al., 2018). Since in our study the occurrence of stunted arbuscules in the *str1* mutant was sufficient to elicit the blumenol accumulation, we propose that arbuscule formation triggers blumenol synthesis. Maintenance of blumenol accumulation in *str1* which only forms shrivelled arbuscules suggests that normal arbuscule formation and function are not required for blumenol accumulation. Instead, the occurrence of foliar blumenol appears to rely either on formation of intraradical colonisation structures or on associated gene signalling pathways initiated by the formation of arbuscules specifically, the expression of which are lost in rice *ccamk* and *pollux* mutants (Gutjahr et al., 2008). A requirement of arbuscule formation and subsequent signalling for blumenol biosynthesis and accumulation would be consistent with the data described here; however, uncoupling cortex colonisation from arbuscule formation would be necessary to unambiguously determine causality, which awaits further assessment.

It has previously been shown that the SL biosynthesis gene *CCD7/D17* is required for blumenol biosynthesis (Vogel et al., 2010). Analysis of foliar and root blumenol accumulation in rice *d27*, *d17* and *d10* mutants demonstrated for the first time that the D27 isomerase is additionally required for blumenol biosynthesis, and confirmed a role for D17 in blumenol synthesis beyond tomato also in rice, whereas D10 is not required at all. Such finding provides strong evidence towards the current model of blumenol biosynthesis (Figure 4A), where D27 facilitates isomerisation required for the stereospecific D17/CCD7 to yield C27 intermediates in the first dedicated step in blumenol biosynthesis (Bruno et al., 2014). Further work is still needed to determine the enzymes involved in later stages of C13 blumenol modification to form AMF‐specific derivatives such as 11‐carboxyblumenol‐C‐glc (You et al., 2023).

To elucidate the potentially differing roles of SL biosynthesis enzymes for their involvement in blumenol biosynthesis, we assessed AM phenotypes in *d27*, *d17* and *d10*. We found that there is greater plasticity in requirement for *D17* and *D10* in symbiosis than previously anticipated (Gutjahr et al., 2012; Kobae et al., 2018) and newly report a requirement for *D27* but with similar variability of colonisation levels as the other SL biosynthetic mutants. While SL deficiency can cause reduced establishment, colonisation progresses to wild‐type levels in all the mutants.

Intriguingly, despite this phenotypic plasticity, we repeatedly observed that *d17* exhibits a more severe colonisation establishment delay than *d27* and *d10*, corroborating previous reports of a more pronounced role for D17/CCD7 in pre‐symbiosis than D10/CCD8 (Kobae et al., 2018). Our findings of maintained wild‐type colonisation in *d27* and *d17*, which are both SL and symbiotic‐blumenol deficient, support that this is independent to a previously proposed role of blumenols, and CCD7, in later stages of symbiosis (López‐Ráez et al., 2015). In fact, consistent with previous findings of normal levels of intraradical colonisation in *ccd1* mutants (Floss et al., 2008; You et al., 2023), we clarify that foliar and root accumulation of blumenol is not required to support intraradical colonisation in rice. Instead, accumulation of blumenol in the shoot might plausibly facilitate systematic signalling of plant fitness in response to root colonisation as proposed earlier (You et al., 2023).

Based on our findings, we provide evidence for a partially shared biosynthetic pathway in SL and blumenol production in rice (Figure 4A). Intriguingly, our data confirm diverging regulation of SL and blumenol synthesis. While SLs are known to be induced prior to engaging with the fungal partner (Volpe et al., 2023), blumenols are strictly associated with intimate cortex invasion. Moreover, while SL synthesis and secretion are regulated by the plant's P_i_‐status and the D14L/SMAX1 signalling pathway, blumenol accumulation is independent of these cues. We therefore favour the hypothesis that distinct fine‐tuned signalling mechanisms drive the production the one or the other apocarotenoid in dedicated cell types during pre‐symbiosis and symbiosis.

## EXPERIMENTAL PROCEDURES

### Plant and fungal material and growth conditions

*Oryza sativa* spp. *japonica* cultivars were used for all experiments. Previously characterised mutants were assessed and included Tos17 insertions (Miyao et al., 2003) in *ccamk‐1* and *pollux‐2* (Gutjahr et al., 2008); a T‐DNA insertion (Sallaud et al., 2004) in *str1‐2* (Gutjahr et al., 2012) in cv. Nipponbare background; tillering dwarf (Ishikawa et al., 2005) SL biosynthesis mutants *d27‐1*(Lin et al., 2009), *d17‐1* and *d10‐1* (Umehara et al., 2008) in cv. Shiokari background; and a T‐DNA insertion (Jeon et al., 2000) in *smax1‐1* in cv. Dongjin background (Choi et al., 2020). Seeds were surface‐sterilised in 70% (v/v with water) ethanol and 3% (v/v with water) sodium hypochlorite before germination on 0.8% bactoagar plates. Seedlings were incubated at 30°C for 4–6 days. Seedlings were then planted in cones (2.5 cm diameter, 12 cm depth) which contained sterile quartz‐sand. Spores of *R. irregularis* (DAOM197198) were extracted from transgenic hairy carrot root cultures and suspended in water to produce inoculum for AM assays. The growth medium was inoculated with 1 ml of either diH_2_O (mock control) or diH_2_O solution containing 100, 300, 500 or 700 spores of *R. irregularis* (DAOM197198), as stated in text. Plants were grown in walk‐in chambers under 12‐h:12‐h light:dark cycle, 28:20°C and 60% relative humidity. All plants were watered three times a week up to 2 wpi with diH_2_O, after which they were fertilised twice weekly using half Hoagland solution containing 25 μm phosphate (P_i_) concentration. Plants in P_i_‐gradient experiment were watered with the same regime but using half Hoagland solution containing 25, 250 or 2500 μm P_i_, respectively.

### Plant harvest

Time of harvest varied and is specified in the main text and figure legends. Plants were removed from cones and washed in diH_2_O. The root system was cut into 2–3 cm pieces, and a representative sample of all root types was taken for gene expression, blumenol metabolic and root colonisation assays. Shoot material was cut into 2–3 cm pieces, and a ~100–200 mg fresh weight sample containing a representative mix of all leaf types was taken for blumenol metabolic analyses described below.

### Microscopic quantification of AM colonisation

Root material was incubated at 95°C for 30 min in KOH and then washed three times in diH_2_O. Root samples were incubated in 1.5 ml of 0.3 m HCl for 20 min. HCl was removed, and 1 ml of 0.1% (w/v) Trypan Blue (Sigma‐Aldrich, St Louis, MO, USA) staining solution was added. Samples were incubated for 5 min at 95°C. Roots were stored in trypan blue or immediately transferred onto slides. To make microscope slides, roots were first washed in 50% (w/v) acidic glycerol. Ten representative roots were transferred onto glass microscope slides using forceps. A drop of 50% (w/v) acidic glycerol was pipetted onto the slide before adding a cover slip. Slides were sealed with varnish to prevent drying. Fungal structures were scored according to a modified gridline intersect method previously described (Paszkowski et al., 2006). Structures are marked as present or absent in the field of vision at 10 equal intervals along each root piece analysis of 10 root pieces per plant, representative of total root length colonisation. Fungal structures scored include extraradical hyphae (EH), intraradical hyphae (IH), hyphopodia (H), arbuscules (A), vesicles (V) and spores (SP).

### Metabolic quantification of blumenol‐C‐glucosides

Representative samples of ~100–200 mg fresh weight of root and shoot pieces were shock‐frozen in liquid nitrogen before grinding to a powder in a GenoGrinder 2000 (SPEX SamplePrep, Metuchen, NJ, USA) for 1‐min intervals at 1000 strokes min^−1^. Fresh weight measurements were used for normalisation. Samples were extracted in MeOH extraction buffer before ultra‐high‐performance liquid chromatography triple quadrupole mass spectrometry (Ultimate 3000 RSLC [Thermo Fisher Scientific, Waltham, MA, USA]; EVO‐Q EliteTM [Bruker, Billerica, MA, USA]) was performed, as described previously (Mindt et al., 2019; Wang et al., 2018).

### Gene expression analysis

For RNA extraction and cDNA synthesis, root tissue was shock‐frozen in liquid nitrogen and lysed using Qiagen Tissue Lyser II (Qiagen, Hilden, Germany) at 30 Hz for 1‐min intervals until material was a fine powder. Samples were extracted by a TRIzol method described previously (Choi et al., 2020). Extracted RNA sample concentration and integrity were assessed on a NanoDrop spectrophotometer (Thermo Fisher Scientific). To check for RNA degradation, samples were examined by 1.5% (w/v) agarose gel electrophoresis. One microgram of RNA was treated with DNaseI (Sigma‐Aldrich), following manufacturer's guidelines, to ensure the absence of gDNA contamination. DNA‐free RNA was then used for First Strand complementary DNA (cDNA) synthesis using SuperScript II Reverse Transcriptase (Invitrogen, Waltham, MA, USA), following manufacturer's guidelines. cDNA produced was quantified by spectrophotometry using a NanoDrop (Thermo Fisher Scientific) and stored at −20°C. For gene expression analysis, cDNA was diluted at 1:12 ratio with ice‐cold diH_2_O. Quantitative Real‐Time (qRT)‐PCR reaction mix with primers of tested efficiency was added to DNA in 96‐ and 384‐well plates. All reactions used a standard PCR cycling programme with the CFX96 Touch Real‐Time PCR detection system (Bio‐Rad, Hercules, CA, USA). All samples were tested in plates alongside diH_2_O negative control and three housekeeping genes: Cyclophilin (CP2, LOC_Os02g02890), Ubiquitin (UbiQ, LOC_Os02g02890) and GAPDH (LOC_Os02g02890). The following primer pairs were used: *OsCP2* fwd 5′‐GTGGTGTTAGTCTTTTTATGAGTTCGT‐3′ rev ACCAAACCATGGGCGATCT, *OsPOLYUBIQUITIN* fwd CATGGAGCTGCTGCTGTTCTAG rev 5′‐CAGACAACCATAGCTCCATTGG‐3′, *OsGAPDH* fwd 5′‐CTGATGATATGGACCTGAGTCTACTTTT‐3′ rev 5′‐CAACTGCACTGGACGGCTTA‐3′, *OsIPS1* fwd 5′‐AAGGGCAGGGCACACTCCACATTATC‐3′ rev 5′‐ATTAGAGCAAGGACCGAAACACAAAC‐3′, *OsAM14* fwd 5′‐GAGAAGTTCCCTGCTTCAAGCA‐3′ rev 5′‐CATATCCCAGATGAGCGTATCATG‐3′. The primers were described previously – *OsIPS1* (Hou et al., 2005, p. 200), *OsCP2*, *OsPOLYUBIQUITIN*, *OsGAPDH* and *OsAM14* (Gutjahr et al., 2008). The *C*_t_ value per gene of interest was quantified against the *C*_t_ values for the three housekeeping genes using a geometric mean calculation. Geometric mean *C*_t_ values were recorded as *C*_t_ normalised to *CP2* and log_10_ (*x* + 1000) transformed for scaling.

### Statistical analysis

All statistical analyses were done with RSTUDIO (http://www.Rproject.org/) using R version 4.2.1. Assumptions of normality and equal variance were analysed using histograms and QQ plots of data and Shapiro–Wilkinson test. Statistical differences between non‐normally distributed data were analysed using the non‐parametric Kruskal–Wallis and *post‐hoc* testing using Fisher's least significant difference at 5% significance level. Pairwise comparisons between genotypes identified statistically different groups, denoted by different letters or asterisks on graphs. Letter colour depicts mock or *R. irregularis* inoculated samples. This was performed using base R and agricolae (v1.3‐5; https://cran.r‐project.org/web/packages/agricolae/agricolae.pdf) package. Blumenol correlation analyses used linear regression of blumenol abundance against % colonisation were performed as described previously (Wang et al., 2018), using base R and stats (R Core Team, 2013) package.

## AUTHOR CONTRIBUTIONS

EKS and UP designed the experiments and wrote the paper. EKS conducted AM experiments and analysed the data. ITB, RH and ACR conducted the metabolite analyses.

## CONFLICT OF INTEREST

The authors declare no conflicts of interest.
